# A Strapdown Interial Navigation System/Beidou/Doppler Velocity Log Integrated Navigation Algorithm Based on a Cubature Kalman Filter

**DOI:** 10.3390/s140101511

**Published:** 2014-01-15

**Authors:** Wei Gao, Ya Zhang, Jianguo Wang

**Affiliations:** 1 College of Automation, Harbin Engineering University, Harbin 150001, China; E-Mail: gaow@hrbeu.edu.cn; 2 Department of Earth and Space Science and Engineering, York University, Toronto, ON M3J 1P3, Canada; E-Mail: jgwang@yorku.ca

**Keywords:** integrated navigation, Beidou, Cubature Kalman filter, asynchronous, information fusion

## Abstract

The integrated navigation system with strapdown inertial navigation system (SINS), Beidou (BD) receiver and Doppler velocity log (DVL) can be used in marine applications owing to the fact that the redundant and complementary information from different sensors can markedly improve the system accuracy. However, the existence of multisensor asynchrony will introduce errors into the system. In order to deal with the problem, conventionally the sampling interval is subdivided, which increases the computational complexity. In this paper, an innovative integrated navigation algorithm based on a Cubature Kalman filter (CKF) is proposed correspondingly. A nonlinear system model and observation model for the SINS/BD/DVL integrated system are established to more accurately describe the system. By taking multi-sensor asynchronization into account, a new sampling principle is proposed to make the best use of each sensor's information. Further, CKF is introduced in this new algorithm to enable the improvement of the filtering accuracy. The performance of this new algorithm has been examined through numerical simulations. The results have shown that the positional error can be effectively reduced with the new integrated navigation algorithm. Compared with the traditional algorithm based on EKF, the accuracy of the SINS/BD/DVL integrated navigation system is improved, making the proposed nonlinear integrated navigation algorithm feasible and efficient.

## 1. Introduction

In modern marine navigation, the strapdown inertial navigation systems (SINS) is widely used due to its advantages of being more compact and autonomous. However, accumulated navigation errors are inevitable in SINS and may become considerably conspicuous in the long-term. Consequently, it is often aided with other sensors, e.g., global positioning system (GPS) and Doppler velocity log (DVL) *etc*. The accuracy of the integrated system can thus be effectively improved owing to the redundancy and complementarity of the measurements [1–3]. Nowadays, the GPS-aided SINS integrated system is the most popular marine navigation system. Besides GPS, GLONASS, Gallileo, and another satellite navigation system named Beidou (BD) is being developed, which can provide precise position information via the double-star positioning theory [4,5]. This study focuses on the SINS/BD integrated system and further integrates DVL into the SINS/BD system to maintain and improve the system accuracy in poor BD or BD denied environments [5,6].

One outstanding feature of BD is that it is an active inquiry-response positioning system. The user's position information is sent to the ground central control system through two satellites and then processed by the ground central control system. Then, the processed information is sent back to the satellites, and finally the estimated user's position is sent to the user by the satellites [7,8]. Accordingly, the signals are transmitted multiple times between the ground receiver and satellites. With the additional processing time of the calculation center, time-delays appear in the user's position. This causes the asynchronous phenomenon in a SINS/BD/DVL integrated navigation system, which will degrade the accuracy of the system. Therefore, an advanced asynchronous algorithm with small computational cost and high accuracy is important for SINS/BD/DVL integrated navigation.

To solve the multi-sensor asynchronous problem, a SINS/Beidou/STAR integrated navigation system based on the federal filtering algorithm was built up [9]. Prior delayed information was recorded to correct the estimated states and their covariance matrix. In [10] an algorithm of weighted covariance for centralized asynchronous fusion (WCCAF), which fused the latest predicted state vector with the existing estimated state vector was proposed. The simulation results showed that the maximal position RMSE was 6 m in 90 s with the proposed method. Although these two methods could dampen the estimation error due to the asynchronization among multiple sensors, both of them are based on Kalman filters, so they are only suitable for linear systems. Since almost all actual systems are nonlinear, nonlinear filters should be used for multi-sensor information fusion [11–18]. In [11] an information fusion algorithm based on the Extend Kalman Filter (EKF) was introduced to solve nonlinear problems in multi-sensor integrated navigation, but the precision is limited because of the Taylor expansion and the EKF needs to calculate the fussy Jacobian matrix which increases the computational load. With the presented algorithm, 80% of errors in estimation are within 16 m in 50 s. The authors of [18] proposed an integrated navigation algorithm based on Unscented Kalman Filter (UKF) which was applied to a SINS/CNS (Celestial Navigation System)/GPS integrated system. In [18], the local UKF was used to estimate the nonlinear integrated system and the federated Kalman filter was used to fuse the predictions of local filters, but in high-dimensional systems, the computation load is still heavy, thus, the filter converges slowly. In 2009, Arasaratnam and Haykin [19] proposed a more accurate nonlinear filtering solution based on a Cubature transform named Cubature Kalman filter (CKF) which can avoid linearization of the nonlinear system by using Cubature point sets to approximate the mean and variance. The third-order accuracy of the system can be achieved with this method. Because of its high accuracy and low calculation load, the CKF is widely used in attitude estimation and navigation [20–22].

In this paper a novel asynchronous algorithm for the SINS/BD/DVL integrated navigation system is proposed on the basis of CKF. Meantime, new nonlinear system and measurement models are also established for the measurements from SINS, BD and DVL. Taking multi-sensor asynchronization into account, a new sampling principle is proposed to make the best use of individual measurements. Even better, CKF can not only reduce the computational complexity, but also improve the accuracy of the navigation solution. The results from simulations showed that the proposed algorithm is superior to the conventional one. The rest of the paper is organized as follows. The description of the error differential equations of the SINS/BD/DVL integrated navigation system and the nonlinear filter named CKF are presented in Section 2. Section 3 shows the new sampling principle and the new asynchronous integrated navigation algorithm. Numerical examples along with specfic analysis are given in Section 4. Section 5 concludes this manuscript.

## 2. Sensor Error Models and Nonlinear CKF

### 2.1. Nonlinear Error Model of SINS

Traditional linear differential equations are obtained under the assumption that the misalignment angles are small, so modeling errors are inevitable due to the nonlinearity of the true error model [3]. To improve the accuracy of the system model, a nonlinear error model of large azimuth misalignment angle for SINS is considered in this paper.

In this paper, *i*, *b*, *e*, *n* and *n*′ denote the inertial coordinate system, the body coordinate system, the earth coordinate system, the navigation coordinate system, and the calculation coordinate system of SINS, respectively. Suppose that *n* can be transformed to *n*′ by turning, *ϕ_z_*, *ϕ_x_* and *ϕ_y_* successively, wherein *ϕ* = [*ϕ_x_ ϕ_y_ ϕ_z_*]*^T^* is the Euler error angle vector, the direction cosine matrix from *n* to *n*′ is $C_{n}^{n^{\prime }}$. Using *sϕ_i_*, *cϕ_i_* (*i* = *x*, *y*, *z*) denote sin(*ϕ_i_*) and cos(*ϕ_i_*), respectively, $C_{n}^{n^{\prime }}$ can be describled as follows:

(1) $$C_{n}^{n^{\prime }}=\left[\begin{array}{ccc} c\phi_{y}c\phi_{z}-s\phi_{y}s\phi_{x}s\phi_{z} & c\phi_{y}s\phi_{z}+s\phi_{y}s\phi_{x}s\phi_{z} & -s\phi_{y}c\phi_{x}\\ -c\phi_{x}s\phi_{z} & c\phi_{x}c\phi_{z} & s\phi_{x}\\ s\phi_{y}c\phi_{z}+c\phi_{y}s\phi_{x}s\phi_{z} & s\phi_{y}s\phi_{z}-c\phi_{y}s\phi_{x}s\phi_{z} & c\phi_{y}c\phi_{x} \end{array}\right]$$

The nonlinear attitude error equation of SINS can be derived as follows:

(2) $$\dot{\phi }=C_{\omega }^{-1}\left[\left(I-C_{n}^{n^{\prime }}\right){\hat{\omega }}_{in}^{n^{\prime }}+C_{n}^{n^{\prime }}\delta \omega_{in}^{n}-C_{n}^{n^{\prime }}\varepsilon^{b}\right]+C_{\omega }^{-1}C_{b}^{n^{\prime }}W_{g}^{b}$$

wherein $C_{b}^{n^{\prime }}$ denotes the direction cosine matrix from *b* to *n*′, *ε^b^* and $W_{g}^{b}$ are the gyro constant drift vector and the zero-mean Gaussian white noise vector, respectively, ${\hat{\omega }}_{in}^{n^{\prime }}$ is the gyro measurement vector, $\omega_{in}^{n}$ is the rotating angular rate vector of *n* relative to *i*, $\delta \omega_{in}^{n}$ is the calculated error vector of $\omega_{in}^{n}$. The gyro measurement vector is equal to ${\hat{\omega }}_{in}^{n}=\omega_{in}^{n}+\delta \omega_{in}^{n}$. *C_ω_* is an intermediate matrix as follows:

(3) $$C_{\omega }=\left[\begin{array}{ccc} \cos\phi_{y} & 0 & -\sin\phi_{y}\cos\phi_{x}\\ 0 & 1 & \sin\phi_{x}\\ \sin\phi_{y} & 0 & \cos\phi_{y}\cos\phi_{x} \end{array}\right]$$

The SINS velocity error equation is given by:

(4) $$\delta {\dot{\nu }}^{n}=C_{b}^{n^{\prime }}\;{\hat{f}}^{b}-C_{b}^{n}\;{\hat{f}}^{b}+C_{b}^{n}\;\delta f^{b}-\left(2\delta \omega_{ie}^{n}+\delta \omega_{en}^{n}\right)\times \left({\hat{\nu }}^{n}-\delta \nu^{n}\right)-\left(2{\hat{\omega }}_{ie}^{n}+{\hat{\omega }}_{en}^{n}\right)\times \delta \nu^{n}+\delta g^{n}$$

wherein *f̂^b^* and *δ f^b^* denote the specific force vector and its corresponding error vector respectively, ${\hat{\omega }}_{ie}^{n}$ is the calculated Earth's rotating angular rate, ${\hat{\omega }}_{en}^{n}$ is the calculated angular rate vector, $\delta \omega_{ie}^{n}$ and $\delta \omega_{en}^{n}$ indicate the error vectors of ${\hat{\omega }}_{ie}^{n}$ and ${\hat{\omega }}_{en}^{n}$ respectively, *ν̂^n^* and *δν^n^* denote velocity measurement vector and its corresponding error vector, *δ g^n^* is the gravity acceleration error, and $C_{b}^{n}=C_{n^{\prime }}^{n}C_{b}^{n^{\prime }}$.

Suppose that *δ f^b^* is composed of the constant bias error ∇*^b^* and the zero-mean Gaussian white noise vector $W_{a}^{b}$. If *δ g^n^* is ignored, Equation (4) can be rewritten as follows:

(5) $$\delta {\dot{\nu }}^{n}=C_{b}^{n^{\prime }}\;{\hat{f}}^{b}-C_{b}^{n}\;{\hat{f}}^{b}+C_{b}^{n}\nabla^{b}-(2\delta \omega_{ie}^{n}+\delta \omega_{en}^{n})\times ({\hat{\nu }}^{n}-\delta \nu^{n})-(2{\hat{\omega }}_{ie}^{n}+{\hat{\omega }}_{en}^{n})\times \delta \nu^{n}+C_{b}^{n}W_{a}^{b}$$

Because both of the gyro and accelerometer errors are composed of a constant error vector and a zero-mean Gaussian white noise vector, their differential equations are:

(6) $$\{\begin{array}{l} {\dot{\varepsilon }}^{b}=0\\ {\dot{\nabla }}^{b}=0 \end{array}$$

The position error equations comprise the longitude error *δλ* and the latitude error *δφ*:

(7) $$\{\begin{array}{ll} \delta \dot{\lambda } & =\delta \varphi \tan\varphi \sec\varphi \frac{\nu_{x}}{{\text{R}}_{N}}+\sec\varphi \frac{\delta \nu_{x}}{{\text{R}}_{N}}\\ \delta \dot{\varphi } & =\frac{\delta \nu_{y}}{{\text{R}}_{M}} \end{array}$$

wherein *R_M_* and *R_N_* are the Earth's radii of the meridian circle and the prime vertical circle, respectively; *λ* and *φ* are the longitude and latitude of a point of interest; *ν_x_* and *ν_y_* are the east and north velocities with their velocity errors *δν_x_* and *δν_y_*, respectively.

### 2.2. Error Model of BD

The location information can be received directly from BD. The major error sources which affect the measurement accuracy of BD are the error of the BD receiver, the track error and the multi-path effect. To focus on the asynchronicity problem of multi-sensor systems, only the clock error of a BD receiver is taken into account here, including the clock bias and the clock frequency drift [6]. Despite the fact that the clock bias consists of constant and random components, only the constant bias is taken into account here for simplification. Normally, one uses Δ*t* and *δ_t_* to denote the clock constant bias and the clock frequency drift. So the shaping filter of the BD receiver's clock error is described as follows:

(8) $$\{\begin{array}{l} \Delta \dot{t}=\delta_{t}\\ {\dot{\delta }}_{t}=-\tau \Delta t+W_{\delta } \end{array}$$

wherein *τ* is the correlation time and *W_δ_* is the white noise.

### 2.3. Error Model of DVL

**Figure 1.**
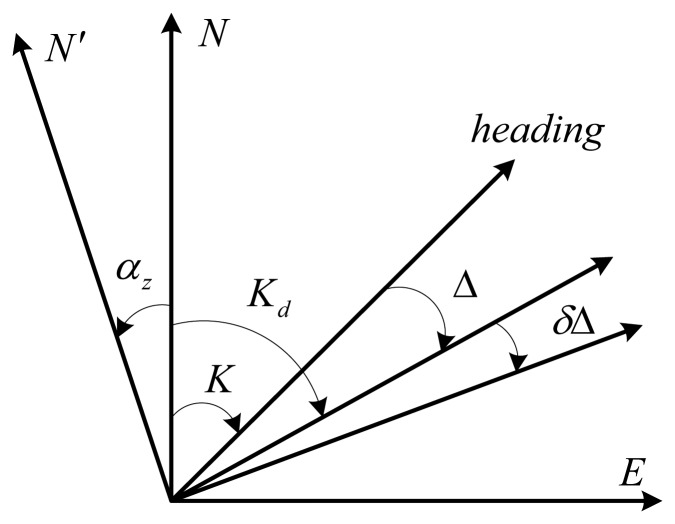
The schematic of the velocity errors measured by the DVL.

The DVL functions as a sensor that measures the frequency shift of an acoustic signal, either transmitted or received by a moving object, which is proportional to the velocity of the moving object [2,23]. It can not only provide high accuracy absolute velocity, but aslo have satisfactory anti-interference performance, hence, DVL is widely deployed in marine navigation systems. The working principle of a DVL is based on the Doppler effect and the principle is described in Figure 1.

In Figure 1, *K* means the true heading, *K_d_* is the heading with the drift angle Δ, the drift of the angle error is denoted by *δ*Δ, and *α_z_* indicates the azimuth misalignment angle. By using $V_{d}^{\prime }$ to denotes the velocity vector measured by the DVL, the following velocity equations are satisfied:

(9) $$V_{d}^{\prime }=\left(1+\delta C\right)\left(V_{d}+\delta V_{d}\right)$$

(10) $$\{\begin{array}{l} V_{dx}^{\prime }=(1+\delta C)\left(V_{d}+\delta V_{d}\right)\sin(K_{d}+\alpha_{z}+\delta \Delta )\\ V_{dy}^{\prime }=(1+\delta C)\left(V_{d}+\delta V_{d}\right)\cos(K_{d}+\alpha_{z}+\delta \Delta ) \end{array}$$

where *δC* indicates the scale factor error, *V_d_* and *δV_d_* denote the true velocity vector and the velocity drift error vector, respectively. $V_{dx}^{\prime }$ and $V_{dy}^{\prime }$ are the components of $V_{d}^{\prime }$. Since both *α_z_* and *δ*Δ are small enough, the Equation (10) can be rewritten as follows:

(11) $$\{\begin{array}{l} V_{dx}^{\prime }=V_{d}\sin K_{d}+V_{d}\sin K_{d}\cdot \left(\alpha_{z}+\delta \Delta \right)+\delta C\cdot V_{d}\sin K_{d}+\delta V_{d}\sin K_{d}\\ V_{dy}^{\prime }=V_{d}\cos K_{d}-V_{d}\sin K_{d}\cdot \left(\alpha_{z}+\delta \Delta \right)+\delta C\cdot V_{d}\cos K_{d}+\delta V_{d}\cos K_{d} \end{array}$$

From Figure 1, one can also obtain:

(12) $$\{\begin{array}{l} \nu_{x}=V_{d}\sin K_{d}\\ \nu_{y}=V_{d}\cos K_{d} \end{array}$$

According to the working principle of the DVL, one can obtain the velocity and the drift angle relative to the seafloor. Thus, the measurement errors include the velocity drift error *δV_d_*, the scale factor error *δC* and the drift angle error *δ*Δ [2,4]. The DVL error model is as follows:

(13) $$\{\begin{array}{l} \delta {\dot{V}}_{d}=-\beta_{d}\delta V_{d}+W_{d}\\ \delta \dot{\Delta }=-\beta_{\Delta }\delta \Delta +W_{\Delta }\\ \delta \dot{C}=0 \end{array}$$

where $\beta_{d}^{-1}$, $\beta_{\Delta }^{-1}$ denote the correlation times of *δV_d_* and *δ*Δ respectively; *W_d_*, *W*_Δ_ are the corresponding white noises.

### 2.4. Cubature Kalman Filter

Consider the following discrete-time nonlinear state-space model:

(14) $$\{\begin{array}{l} x_{k}=f\left(x_{k-1}\right)+W_{k-1}\\ z_{k}=h\left(x_{k}\right)+\eta_{k} \end{array}$$

wherein *x_k_* and *z_k_* are the state vector and the measurement vector at time *k*, respectively; *f*(·) and *h*(·) are specific known nonlinear functions; and *W_k_*_−1_ and *η_k_* are the noise vectors from two independent zero-mean Gaussian processes with their covariance matrices *Q_k_*_−1_ and *R_k_*, respectively.

CKF is proposed to solve this nonlinear filtering problem on the basis of the spherical-radial cubature criterion. CKF first approximates the mean and variance of probability distribution through a set of 2*N* (*N* is the dimension of the nonlinear system) Cubature points with the same weight, propagates the above cubature points through the nonlinear functions, and then calculates the mean and variance of the current approximate Gaussian distribution by the propagated cubature points [19].

A set of 2*N* Cubature points are given by [*ξ_i_*, *ω_i_*], where *ξ_i_* is the *i*-*th* cubature point and *ω_i_* is the corresponding weight:

(15) $$\{\begin{array}{l} \xi_{i}=\sqrt{N}{\left[1\right]}_{i}\\ \omega_{i}=\frac{1}{2N} \end{array}$$

wherein *i* = 1, 2,…2*N*.

Under the assumption that the posterior density at time *k*−1 is known, the steps involved in the time-update and the measurement-update of CKF are summarized as follows [19]:

Time-update:
  $$\begin{array}{c} P_{k-1|k-1}=S_{k-1|k-1}S_{k-1|k-1}^{T}\\ X_{i,k-1|k-1}=S_{k-1|k-1}\xi_{i}+{\hat{x}}_{k-1|k-1}\\ x_{i,k|k-1}^{*}=f\left(X_{i,k-1|k-1}\right)\\ {\hat{x}}_{k|k-1}=\frac{1}{2N}\overset{2N}{\underset{i=1}{\text{∑}}}X_{i,k|k-1}^{*}\\ P_{k|k-1}=\frac{1}{2N}\sum_{i=1}^{2N}X_{i,k|k-1}^{*}X_{i,k|k-1}^{*T}-{\hat{x}}_{k|k-1}{\hat{x}}_{k|k-1}^{T}+Q_{k-1} \end{array}$$
Measurement-update:
  $$\begin{array}{c} P_{k|k-1}=S_{k|k-1}S_{k|k-1}^{T}\\ X_{i,k|k-1}=S_{k|k-1}\xi_{i}+{\hat{x}}_{k|k-1}\\ Y_{i,k|k-1}=h\left(X_{i,k|k-1}\right)\\ {\hat{y}}_{k|k-1}=\frac{1}{2N}\overset{2N}{\underset{i=1}{\text{∑}}}Y_{i,k|k-1}\\ P_{k|k-1}^{zz}=\frac{1}{2N}\overset{2N}{\underset{i=1}{\text{∑}}}Y_{i,k|k-1}Y_{i,k|k-1}^{T}-{\hat{y}}_{k|k-1}{\hat{y}}_{k|k-1}^{T}+R_{k}\\ P_{k|k-1}^{xz}=\frac{1}{2N}\overset{2N}{\underset{i=1}{\text{∑}}}X_{i,k|k-1}Y_{i,k|k-1}^{T}-{\hat{x}}_{k|k-1}{\hat{y}}_{k|k-1}^{T} \end{array}$$

With the new measurement vector *z_k_*, the estimated of the state vector *x̂_k_*_∣_*_k_* and its covariance matrix *P_k_*_∣_*_k_* at time *k* can be obtained by the following equations:

$$\begin{array}{c} K_{k}=P_{k|k-1}^{xz}{\left(P_{k|k-1}^{zz}\right)}^{-1}\\ {\hat{x}}_{k|k}={\hat{x}}_{k|k-1}+K_{k}\left(z_{k}-{\hat{z}}_{k|k-1}\right)\\ P_{k|k}=P_{k|k-1}-K_{k}P_{k|k-1}^{zz}K_{k}^{T} \end{array}$$

wherein *K_k_* is the filter gain at time.

CKF uses cubature rule and 2*N* cubature point sets [*ξ_i_*, *ω_i_*] to compute the mean and variance of probability distribution without any linearization of a nonlinear model. Thus, the modeling can reach the third-order or higher. Furthermore, this filtering solution does not demand Jacobians and Hessians so that the computational complexity will be alleviated to a certain extent.

## 3. Novel Nonlinear Integration Algorithm for Nonlinear SINS/BD/DVL Based on CKF

### 3.1. Nonlinear Model of SINS/BD/DVL

The nonlinear model for a SINS/BD/DVL integrated navigation system is established under the large azimuth misalignment angle in this paper. Considering the following error states: the longitude error *δλ*, the latitude error *δφ*, the east velocity error *δν_x_*, the north velocity error *δν_y_*, the Euler angle errors *ϕ_x_*, *ϕ_y_* and *ϕ_z_*, the accelerometer zero-biases ∇*_x_*, ∇*_y_*, the constant gyro drifts *ε_x_*, *ε_y_*, *ε_z_*; the clock constant bias Δ*t* and the clock frequency drift *δ_t_* of the BD clock error, the velocity drift error *δV_d_*, the scale factor error *δC* and the drift angle error *δ*Δ of DVL, the state vector is built up as follows:

$$X={\left[\begin{array}{ccccccccccccccccc} \delta \varphi & \delta \lambda & \delta \nu_{x} & \delta \nu_{y} & \phi_{x} & \phi_{y} & \phi_{z} & \nabla_{x} & \nabla_{y} & \varepsilon_{x} & \varepsilon_{y} & \varepsilon_{z} & \Delta t & \delta_{t} & \delta V_{d} & \delta \Delta & \delta C \end{array}\right]}^{T}$$

The corresponding state equation is written as:

(16) $${\dot{X}}_{k}=f(X_{k-1})+W_{k-1}$$

The state function *f*(·) can be obtained from Equations (1)–(13) and [3]. Futher, the process noise vector is given by:

$$W={\left[\begin{array}{ccccccccccc} 0_{1\times 2} & W_{ax} & W_{ay} & W_{gx} & W_{gy} & W_{gz} & 0_{1\times 6} & W_{\delta } & W_{d} & W_{\Delta } & 0 \end{array}\right]}^{T}$$

wherein *W_ax_* and *W_ay_* are the white noises of accelerometer; *W_gx_*, *W_gy_* and *W_gz_* are the white noises of gyroscopes drifts; *W_δ_* is the white noise; *W_d_*, *W*_Δ_ are the white noises of *δV_d_* and *δ*Δ, respectively.

To solve the problem of asynchronism, a new method is proposed to establish the measurement equations. The multi-sensor measurements can be pre-processed separately. Then, the central fusion blends all of the pre-processed data to obtain the optimal state vector. Here, the measurements are divided into two groups: pseudo-ranges and pseudo-range rates as the measurements for the SINS/BD filter, and the velocity errors as measurements for the SINS/DVL filter.

The measurement equation for the SINS/BD filter is [8]:

(17) $$\{\begin{array}{ll} \delta \rho_{i} & =\delta \varphi \left(R_{N}\left(-e_{i1}\sin\varphi \cos\lambda -\sin\varphi \cos\lambda \right)+e_{i3}R_{N}{\left(1-f\right)}^{2}\cos\varphi \right)+\delta \lambda \left(R_{N}\left(e_{i2}\cos\varphi \cos\lambda -e_{i1}\cos\varphi \cos\lambda \right)\right)+\nu_{c}\cdot \Delta t+\eta_{1,ip}\\ \delta {\dot{\rho }}_{i} & =\delta \nu_{x}\left(-e_{i1}\sin\lambda +e_{i2}\cos\lambda \right)+\delta \nu_{y}\left(-e_{i1}\sin\varphi \cos\lambda -e_{i2}\sin\lambda \cos\varphi +e_{i3}\cos\varphi \right)+\nu_{c}\cdot \delta +\eta_{1,i\dot{\rho }} \end{array}$$

wherein *i* = 1,2,3,4 is the number of satellites, *δρ_i_* and *δρ̇_i_* are the pseudo-range residual and the pseudo-range rate residual between SINS and BD receiver, respectively, *ν_c_* is the velocity of light, *e_i_*_1_, *e_i_*_2_, *e_i_*_3_, are the direction cosine from the user to the *i*-*th* satellite, *η*_1,_*_iρ_* and *η*_1,_*_iρ̇_* are the measurement noise vectors.

The velocity error measurements between the SINS and the DVL are as follows:

(18) $$\{\begin{array}{ll} Z_{2,x} & =-\delta \nu_{x}+\nu_{y}\phi_{z}+\nu_{y}\delta \Delta +\nu_{x}\delta C+\delta V_{d}\sin K_{d}+\eta_{2,x}\\ Z_{2,y} & =\delta \nu_{y}+\nu_{x}\phi_{z}+\nu_{x}\delta \Delta -\nu_{y}\delta C-\delta V_{d}\cos K_{d}+\eta_{2,y} \end{array}$$

wherein *η*_2,_*_x_*, *η*_2,_*_y_* are the DVL measurement noises.

### 3.2. Nonlinear Integration Navigation Algorithm Based on CKF

In this subsection, a CKF-based novel nonlinear algorithm is structured to solve the asynchronicity problem. In general, the smaller the sampling interval one uses, the higher system accuracy one can achieved, but accompanied with a larger calculation burden. A proper sampling interval should be designed accordingly. Now, a new sampling principle is presented. If the sampling interval of SINS, BD and DVL are *T*_1_, *T*_2_ and *T*_3_ respectively, the greatest common divisor (GCD) of *T*_1_, *T*_2_ and *T*_3_ is denoted as *GCD*(*T*_1_,*T*_2_,*T*_3_). Thus, the sampling interval of the integrated navigation system Δ*T* is set as below:

$$\Delta T=\frac{\min(T_{1},T_{2},T_{3})}{GCD(T_{1},T_{2},T_{3})}$$

**Figure 2.**
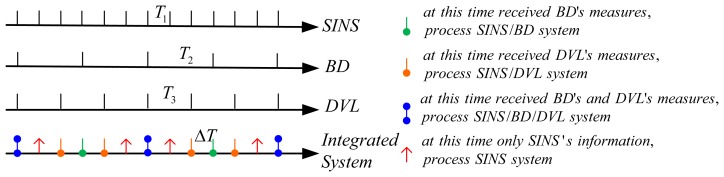
The sampling principle of SINS/BD/DVL integrated navigation system.

Using this sampling criterion Δ*T* is the maximal sampling interval which can sample all of sensors' measurements. So the system accuracy can be improved without the expense of calculation burden. The sampling principle of the SINS/BD/DVL integrated navigation system is described as Figure 2.

(a) If only the measurements from SINS and BD are available at time *k*, the local SINS/BD states can directly be estimated using CKF. For more details on this please refer to Section 2.2. Based on the locally estimated state vector *X̂*_1_ and its covariance matrix *P*_1_, the state vector of the SINS/BD/DVL integrated navigation system at time *k* can be estimated as follows:
  $${\hat{X}}_{f,k}={\hat{X}}_{1,k}$$
(b) Similarly, if only the measurements from SINS and DVL are available at time *k*, the local SINS/DVL states are also directly estimated using CKF. From the locally estimated state vector *X̂*_2_ and its covariance matrix *P*_2_, the state vector of the SINS/BD/DVL integrated navigation system at time *k* can be determined by
  $${\hat{X}}_{f,k}={\hat{X}}_{2,k}$$
(c) If all measurements from SINS, BD and DVL are available at time *k*, the local states for SINS/BD and SINS/DVL are estimated using their own CKF respectively. Thus, one can first estimate the local state vectors *X̂*_1_, *X̂*_2_ and their covariance matrixes *P*_1_, *P*_2_, and then combine the locally estimated state vectors by sensor nodes:
  $${\hat{X}}_{f,k}=D_{1}{\hat{X}}_{1}+D_{2}{\hat{X}}_{2}$$
  wherein *D*_1_ and *D*_2_ are the corresponding weighting matrices for both of the subsystem: SINS/BD and SINS/DVL. Suppose that the sensors are independent, the individual suboptimal estimations of the state vectors can be obtained. After the minimum variance principle, the weighting matrices can be determined, which is explained in details in [12]. Finally, the weighted state vector of the SINS/BD/DVL integrated navigation system at time *k* is deduced as:
  $${\hat{X}}_{f,k}=P_{f,k}\sum_{i=1}^{2}P_{f,k}^{-1}{\hat{X}}_{i,k}$$
  with:
  $$P_{f,k}^{-1}=\sum_{i=1}^{2}P_{i,k}^{-1}$$
(d) If no measurement is available at time *k*, the time-update can be performed to predict the state vector from the previous time. Thus, the state vector of the SINS/BD/DVL integrated navigation system is:
  $${\hat{X}}_{f,k}=\hat{X}(k|k-1)$$

**Figure 3.**
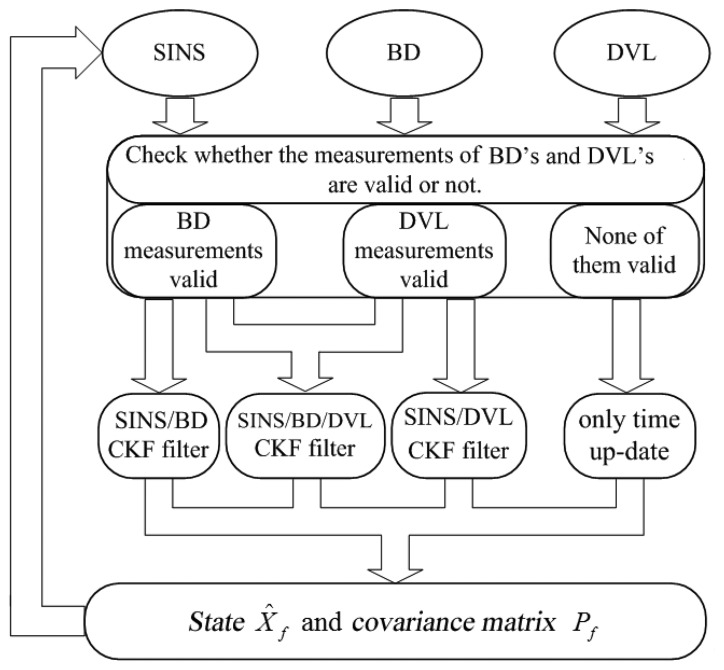
Flow chart of novel algorithm based on CKF.

Figure 3 illustrates the proposed nonlinear algorithm based on CKF.

The solution accuracy of the SINS/BD/DVL integrated navigation system can be improved enormously via CKF whilst the asynchronous problem is solved by this method. Besides, the computational cost of the BD control system of the ground center can also be reduced by using this sampling principle.

## 4. Simulations and Results

**Figure 4.**
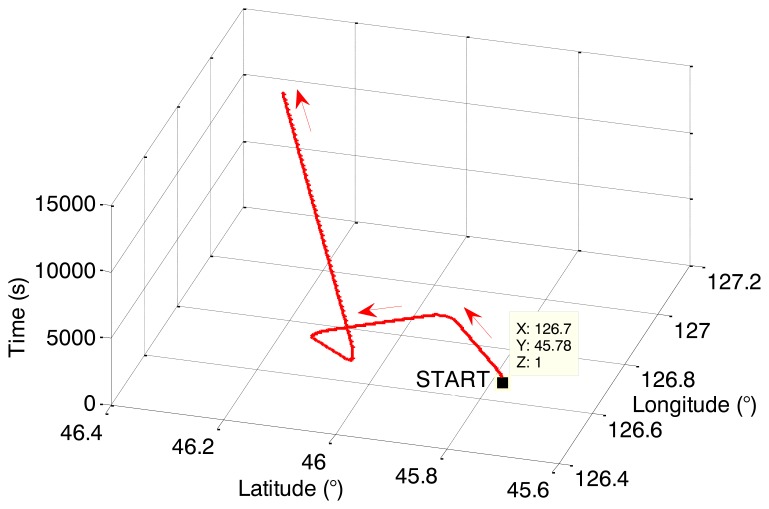
Sailing track of the marine vehicle.

Simulations were performed in this work. Their results are presented in this section. Suppose that the swing dynamic model of a marine vehicle is given by:

$$\{\begin{array}{l} \psi =\psi_{m}\sin(\omega_{\psi }t)+\psi_{k}\\ \theta =\theta_{m}\sin(\omega_{\theta }t)+\theta_{k}\\ \gamma =\gamma_{m}\sin(\omega_{\gamma }t)+\gamma_{k} \end{array}$$

where *θ*, *γ* and *ψ* are pitch, roll and yaw angles, respectively; the swing amplitudes were set up as *θ_m_* = 5°, *γ_m_* = 3°, and *ψ_m_* = 8°; the swing periods were *T_θ_* = 8 *s*, *T_γ_* = 6 *s*, *T_ψ_* = 10 *s*; and the initial attitudes were *θ_k_* = *γ_k_* = 0°, *ψ_k_* = 30°. The vehicle's motion states are listed in Table 1. The total time of each simulation was 10,800 s, and the sailing track of the vehicle is shown as in Figure 4.

The initial conditions of different sensors are presented as follows:

(1) The initial latitude and longitude: *φ* = 45.7796°, *λ* = 126.6705°; their errors: *δφ* = 0.5°, *δλ* = 0.5°;
(2) The initial velocity components: *ν_x_* = 0, *ν_y_* = 0; their errors: *δν_x_* = 0.8 *m*/*s*, *δν_y_* = 0.8 *m*/*s*;
(3) The acceleration due to the gravity: *g*_0_ = 9.7805 *m*/*s*^2^;
(4) The initial misalignment angles: *ϕ_x_* = 1°, *ϕ_y_* = 1°, *ϕ_z_* = 5°;
(5) The SINS gyro constant drifts: *ε_x_* = *ε_y_* = *ε_z_* = 0.01°/*h*;
(6) The SINS gyro random noises: *W_gx_* = *W_gy_* = *W_gz_* = 0.005°/*h*;
(7) The SINS accelerometer constant biases: ∇*_x_* = ∇*_y_* = 10^−4^ *g*_0_;
(8) The SINS accelerometer random noises: *W_ax_* = *W_ay_* = 10^−5^ *g*_0_;
(9) The constant bias of the BD clock error: Δ*t* = 30 *m*;
(10) The frequency drift of the BD clock error: *δ_t_* = 0.01 *m*/*s*;
(11) The correlation time: *τ* = 30 *min*;
(12) The DVL velocity drift error: *δV_d_* = 0.05 *m*/*s*;
(13) The DVL scale factor error: *δC* = 10^−4^;
(14) The DVL drift angle error: *δ*Δ = 1′;
(15) The correlation times of *δV_d_* and *δ*Δ: $\beta_{d}^{-1}=5\;\text{min}$, $\beta_{\Delta }^{-1}=15\;\text{min}$.

**Figure 5.**
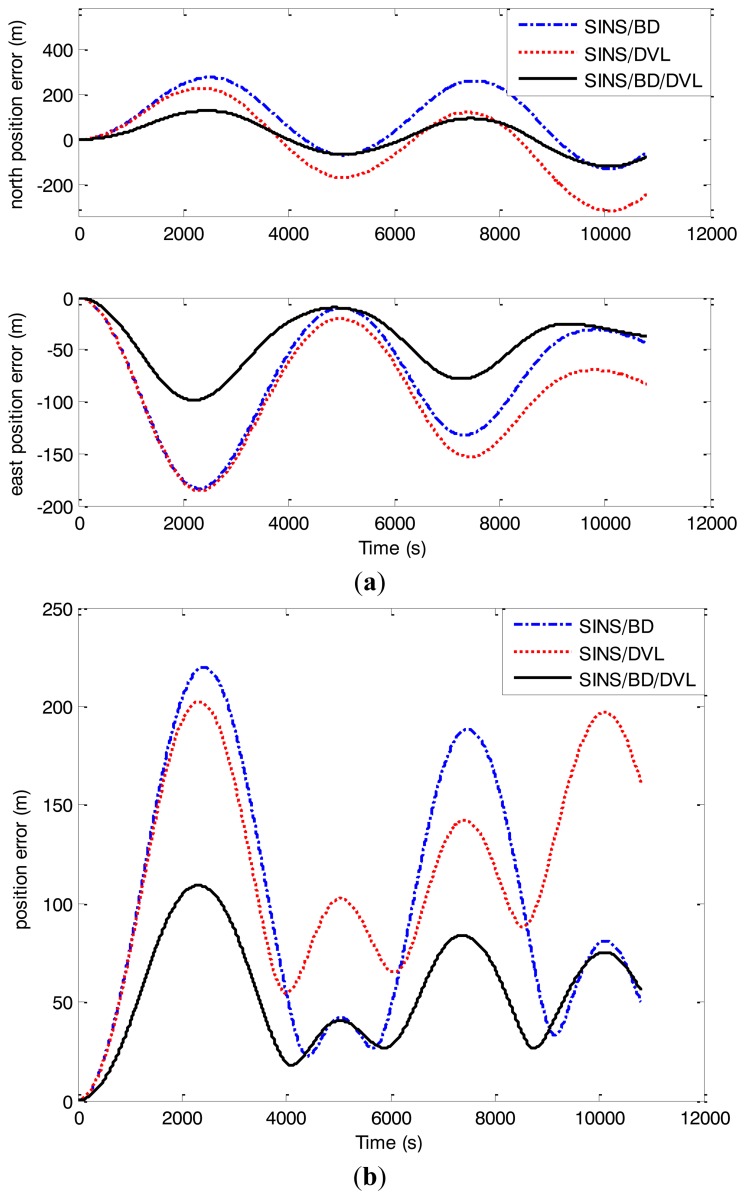
(**a**) The north and east position errors compared with the ones from the individual subsystems; (**b**) The position errors compared with the ones from the individual subsystems.

Under the same simulation conditions, the nonlinear algorithm based on CKF was used to estimate state vectors for the SINS/BD/DVL integrated navigation system. The solution was compared with the CKF solution only using the measurements from SINS/BDor from SINS/DVL. Assume the sampling intervals of BD and DVL are 0.5 s and 0.1 s, respectively, while the sampling interval of the fusion center is 0.05 s. First, the alignment lasted 15 min, and then the navigation was performed. The simulation results are presented in Figure 5 and Table 2. Here the north position error, the east position error and the position error are used to describe the performance of the simulation results in which the location error is as follows:

$$\text{position error}=\sqrt{{\left(\text{north position error}\right)}^{2}+{\left(\text{east position error}\right)}^{2}}$$

Figure 5 and Table 2 show that the north position error, the east position error and the position error from the SINS/BD/DVL integrated solution were much smaller than the errors from the subsystems: SINS/BD and SINS/DVL respectively. Besides, the position error converged rapidly with the proposed algorithm. By using the redundant and complementary measurements from the SINS/BD/DVL integrated navigation system, the novel algorithm can reduce the impact of the asynchronous problem. Thus, the position error can be decreased availably, and the navigation accuracy can be increased significantly. Since it was assumed that all sensors were independent in this research, the estimation results were suboptimal. The equipment errors, such as the gyro drifts, the accelerometer biases, and the misalignment angles, can also bring errors to the navigation solution. Considering the above reasons, the delivered results are acceptable and reasonable.

**Figure 6.**
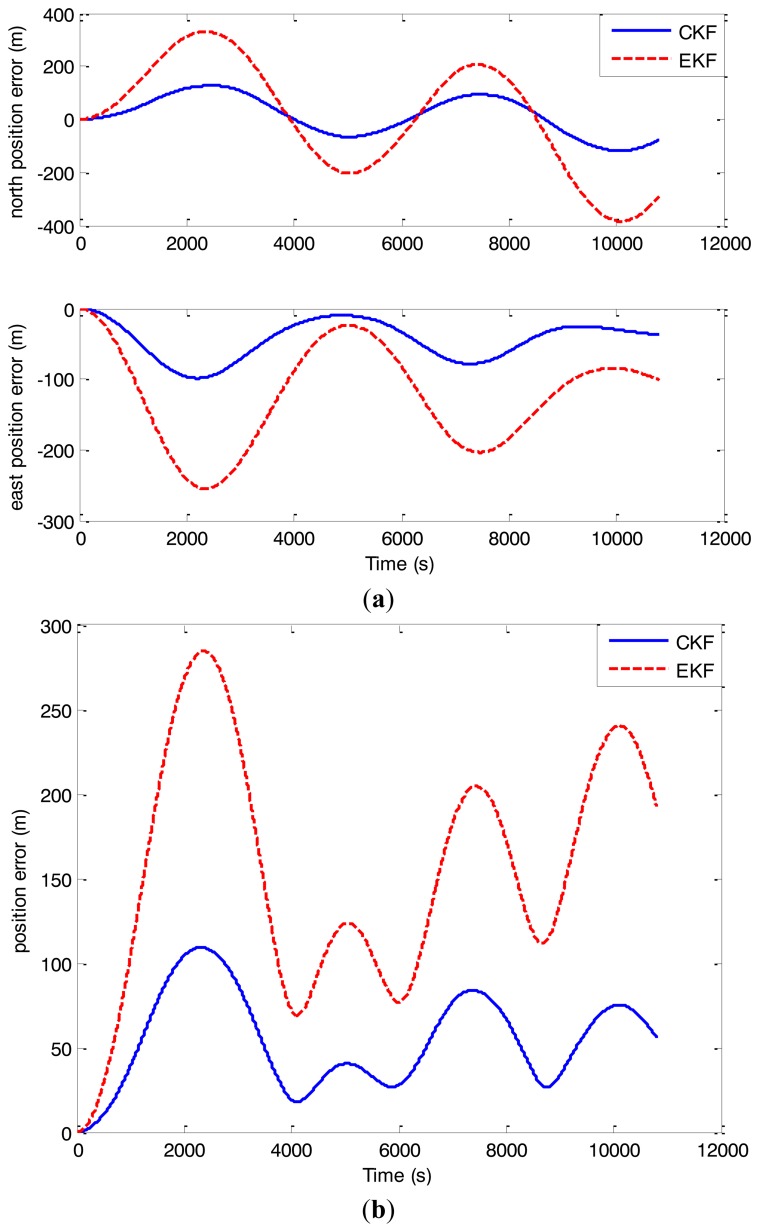
(**a**) The north and east position errors compared with the ones from the traditional algorithm; (**b**) The position errors compared with the ones from the traditional algorithm.

To prove the superiority of the proposed nonlinear asynchronous fusion algorithm based on CKF, another simulation was carried out with the traditional fusion algorithm based on EKF introduced in [11]. The simulation conditions were the same as indicated above. The simulation results are given in Figure 6 and Table 3.

As can be seen from Figure 6 and Table 3, compared with the traditional nonlinear fusion method based on EKF, the north position error, the east position error and the position error of the SINS/BD/DVL integrated navigation system are smaller with the new algorithm based on CKF. With the traditional method based on EKF the maximal position error was about 284 m as the one with the proposed integration algorithm was nearly 109 m. That is, the position error was decreased by 61.6%. As CKF uses cubature rule and 2*N* cubature point sets [*ξ_i_*, *ω_i_*] to compute the mean and variance of probability distribution without any linearization of a nonlinear model, the filtering accuracy can be improved significantly. Hence, the higher navigation accuracy can be obtained based on CKF.

## 5. Conclusions

In this manuscript, a novel nonlinear integrated navigation algorithm based on CKF was proposed in order to solve the multi-sensor asynchronicitybproblem and reduce the high calculation load of the SINS/BD/DVL integrated navigation system. The main focus of this work was on establishing of a nonlinear system model and proposing of a new sampling principle to take multi-sensor asynchronism into account. The superiority of CKF was analyzed theoretically for the situation with the nonlinear system and measurement models. To verify the new navigation algorithm, numerical simulations were carried out. The results showed that the proposed nonlinear fusion algorithm based on CKF cannot only solve the asynchronicity problem of the SINS/BD/DVL integrated navigation system, but also significantly improve the navigation accuracy of the nonlinear system without imposing any additional calculation burden. However, under the assumption made in this study that all sensors in the integrated system were independent, the fusion results were suboptimal. Our future work will focus on a fusion algorithm that is suitable for multi-sensor asynchronous systems with the correlated noises.

## Figures and Tables

**Table 1.** Motion states of the marine vehicle.

**Motions States**	**Time (*s*)**	**Acceleration (*m*/*s*^2^)**
1. Mooring	0–300	*a_x_* = *a_y_*= 0
2. Accelerated motion	300–620	*a_x_* = 0.025, *a_y_* = 0.035
3. Uniform motion	620–1,620	*a_x_* = *a_y_*= 0
4. Accelerated motion	1,620–2,100	*a_x_* = −0.04, *a_y_* = 0.005
5. Uniform motion	2,100–3,100	*a_x_* = *a_y_*= 0
6. Accelerated motion	3,100–3,700	*a_x_* = 0.007, *a_y_* = −0.035
7. Uniform motion	3,700–5,200	*a_x_* = *a_y_*= 0
8. Accelerated motion	5,200–6,200	*a_x_* = 0.018, *a_y_* = 0.015
9. Uniform motion	6,200–10,800	*a_x_* = *a_y_*= 0

**Table 2.** Simulation Results with different sensor data.

**Different Sensor Data**	**Maximal Errors (*m*)**		

	**North Position Error**	**East Position Error**	**Position Error**
SINS/BD	275.1	−183.4	219.8
SINS/DVL	−314.5	−185.9	202.3
SINS/BD/DVL	−118.6	−98.7	109.1

**Table 3.** Simulation results with different filters.

**Different Filters**	**Maximal Errors (*m*)**		

	**North Position Error**	**East Position Error**	**Position Error**
EKF	−384.4	−255	284.5
CKF	−118.6	−98.7	109.1
